# Differential Response of Stem and Leaf Oil Yield and Main Quality of *Cymbopogon citratus* (DC.) Stapf to Different Radiation Intensities

**DOI:** 10.1002/fsn3.71226

**Published:** 2025-11-23

**Authors:** Ling Xu, Nan Lu, Zhuo Feng, ZiWei Ning, Chun Xie, YanLi Huang

**Affiliations:** ^1^ College of Tropical Crops Yunnan Agricultural University Pu'er Yunnan China

**Keywords:** lemongrass, organ‐specific differences, physico‐chemical indicators, radiation intensity, volatile components content

## Abstract

*Cymbopogon citratus* (DC.) Stapf (lemongrass), a globally significant medicinal and aromatic plant, is widely used in food and pharmaceutical industries owing to its bioactive essential oil. However, conventional whole‐plant extraction methods face challenges such as inconsistent oil quality and suboptimal stability. To elucidate the effects of cultivation environments and plant tissues on oil quality, this study categorized lemongrass plants into shaded and natural light exposure groups. Essential oils were extracted from leaves and stems using steam distillation, followed by compositional analysis using Headspace Solid‐Phase Microextraction (HS‐SPME) coupled with Gas Chromatography–Mass Spectrometry (GC–MS). The investigation focused on how varying radiation intensities influenced physicochemical properties and volatile components of essential oils from various plant parts. Results demonstrated that both physicochemical properties and volatile compositions of lemongrass essential oils exhibited minimal sensitivity to variations in radiation intensity treatments. Leaf‐derived essential oil exhibited a significantly higher yield than stem oil by 54.48%, but their refractive indices and chromatic aberration values did not differ substantially. Leaf oil contained markedly elevated levels of heterocyclic compounds, ethers, and aldehydes than those in stem oil by 14.61%, 10.83%, and 1.57%, respectively, while hydrocarbons were lower by 6.1%. The primary volatile constituents characterizing lemongrass quality—geraniol and citral (specifically 2,6‐octadienal, 3,7‐dimethyl‐, (E)‐)—were significantly more abundant in the leaves than in the stems by 8.64% and 7.20%, respectively (*p* < 0.01). Therefore, lemongrass essential oil extracted from leaves cultivated under natural light conditions demonstrated the highest oil yield and superior quality attributes. The integration of natural light cultivation with selective leaf harvesting proposes a promising strategy to enhance lemongrass oil quality. This methodology establishes a theoretical basis for advancing downstream processing techniques and developing high‐value products derived from lemongrass essential oil.

## Graphical abstract

Lemongrass leaf oil quality exceeds stems, featuring higher key component content. Natural light rather than shading maintain essential oil physicochemical properties. Cultivating and harvesting leaf under natural light can enhance essential oil quality.

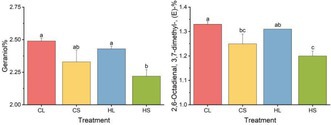

## 1 Introduction

*Cymbopogon citratus* (DC.) Stapf (Lemongrass), a perennial herbaceous plant of the family Poaceae (Ashaq et al. 2024), is widely distributed in tropical regions, including India, Indonesia, Sri Lanka, and Madagascar (Ekpenyong et al. 2014; Shah et al. 2011). Lemongrass is abundant in essential oils and exhibits flavor‐enhancing, antimicrobial, antioxidant, and free‐radical scavenging properties (Kiani et al. 2022; Vázquez‐Fresno et al. 2019), these properties enable it to serve as a traditional flavor enhancer and natural preservative in tropical and subtropical regions. Its essential oil has demonstrated versatile industrial applications, including use in food processing, pharmaceutical products, biocontrol systems, and cosmetic manufacturing (Charlotte et al. 2024; Tazi et al. 2024; Zhong et al. 2022). The expanding utilization of its bioactive compounds correspond to the growing market demand, which underscores its significant commercial potential and research value.

Biochemical studies have demonstrated that the diversity and concentration of bioactive compounds in lemongrass essential oil are key indicators for assessing its quality. Notably, nodal oil cell layers in the stems synthesize high concentrations of monoterpenoids, such as citral (45%–80%), through the mevalonic acid (MVA) pathway, these compounds the primary components of lemongrass essential oil (Ashaq et al. 2024). Monoterpenes including linalool, d‐limonene, *cis*‐ocimene, and *trans*‐ocimene, as along with sesquiterpenes such as caryophyllene and γ‐cadinene, constitute the key chemical constituents responsible for lemongrass' distinctive fresh‐cool aroma. Furthermore, these terpenoid compounds exhibit diverse biological functions, including gastro‐protective and diuretic effects, anemia prevention, and antidiarrheal or analgesic properties (Ekpenyong et al. 2014; Kiani et al. 2022; Shah et al. 2011). At the same time, they confer lemongrass with antioxidant, anti‐inflammatory, and antimicrobial characteristics (Shah et al. 2011; Vázquez‐Fresno et al. 2019). Lemongrass ranks among the most polyphenol‐rich species within the Cymbopogon genus, with its high tannin content significantly enhancing antioxidant properties through potent free radical scavenging capacity (Kiani et al. 2022).

As a dual‐purpose plant with both food and medicinal applications, lemongrass is abundant in flavonoid compounds, such as quercetin and kaempferol. These phytochemicals form a synergistic antioxidant network, which demonstrates critical efficacy in mitigating oxidative stress, mediating anti‐inflammatory responses, and providing cardiovascular protection. Concurrently, this system facilitates free radical neutralization, reduces oxidative cellular damage, and delays aging processes (Kiani et al. 2022; Tazi et al. 2024).

Studies have demonstrated that habitat heterogeneity exerts significant regulatory effects on crop yield and quality. Specifically, appropriate shading can enhance the leaf area index, optimize the soil microenvironment, and improve photosynthetic efficiency, ultimately enhancing the accumulation of volatile compounds in *Vanilla pandanus* plants (Zhong et al. 2022). Conversely, shading in mint cultivation significantly inhibits the activity of key enzymes involved in secondary metabolic pathways, such as terpene synthases and esterases. This inhibition not only reduces the synthesis of volatile aromatic components but also induces morphological alterations, including leaf thinning and reduce photosynthetic capacity (Charlotte et al. 2024). In contrast, lemongrass exhibits superior environmental adaptability—demonstrating optimal growth and essential oil accumulation under full‐light conditions while maintaining tolerance to low‐light stress (Ashaq et al. 2024; Sharma et al. 2021). This photo adaptive plasticity offers significant opportunities for its understory cultivation and industrial expansion. Although current research has preliminarily elucidated the relationship between radiation intensity and lemongrass quality, the specific mechanisms through which varying shading intensities influence key quality parameters, such as essential oil composition and antioxidant activity, remain incompletely understood. A systematic elucidation of the interactions between habitat factors and a secondary metabolism in lemongrass would not only provide theoretical foundation for optimizing cultivation environments but also be of substantial practical value for sustainable industry development.

Under traditional utilization practices, lemongrass has been utilized primarily in its whole‐plant form for industrial applications, including culinary uses and crude citronella oil extraction. As research on plant resource utilization advances, tissue‐specific quality variations in crops have garnered increasing scientific attention. Previous studies have revealed significant organ‐specific compositional disparities in various medicinal plants. For instance, *Byttneria herbacea* Roxb. exhibits significant differences in bioactive compound profiles between leaf and root tissues, leaves demonstrate substantially higher concentrations of total tannins (TAC), total flavonoids content (TFC), and total alkaloids compared to roots (Sharma et al. 2021). Similarly, significant variations in essential oil content have been observed in *Rosmarinus officinalis* , where flowers exhibit the highest oil yield (0.33%–0.35%), while stems and leaves show lower yields (0.26%–0.32%) (Ilić et al. 2022). However, it remains unclear whether similar organ‐specific differences exist in lemongrass and how such variations influence its essential oil quality. Therefore, investigating the optimal habitat conditions for lemongrass cultivation and systematically evaluating tissue‐specific quality disparities are imperative for advancing its value‐added utilization and expanding its industrial applications.

This study conducted field experiments to investigate the effects of natural light versus shading on the yield, physicochemical properties, and volatile profiles of essential oils extracted from lemongrass leaves and stems, and to characterize the initial quality attributes of Chinese lemongrass in Yunnan Province. This study can support the development of graded essential oil extraction protocols and precision utilization systems grounded in habitat regulation and quality assessment by elucidating the interaction mechanisms between growth environments and part‐specific quality attributes. The findings of this study will provide critical insights into optimizing cultivation practices and enhancing the commercial value of lemongrass‐derived products.

## 2 Materials and Methods

### 2.1 Experimental Methods

#### 2.1.1 Introduction to the Experimental Site

The experiment was conducted at the Comprehensive Experimental Farm of the College of Tropical Crops, Yunnan Agricultural University, located in Pu'er City, Yunnan Province, China (102.757° E, 25.135° N; 1303 m a.s.l.). The site experiences a subtropical monsoon climate characterized by year‐round frost‐free conditions, with a mean annual temperature ranging from 15°C to 20.3°C and an annual frost‐free period exceeding 315 days. Precipitation levels vary between 1100 and 2380 mm per annum. The soil type is classified as lateritic red earth.

#### 2.1.2 Experimental Design

The experiment employed a randomized block design with two treatments: natural light exposure (C) and shaded conditions (H). Lemongrass tissues were separately harvested from leaves (L) and stems (S) under these radiation regimes, generating four distinct sample groups labeled as HL (shaded leaves), HS (shaded stems), CL (natural light leaves), and CS (natural light stems). Each treatment was replicated three times, with experimental plots measuring 10 m^2^ (4 m × 2.5 m). Plants were cultivated at a spacing of 50 cm × 50 cm between rows and individuals.

#### 2.1.3 Experiment Management

Lemongrass was propagated by the division on April 27, 2022. The shading treatment involved installing black shading nets (60% light reduction) over experimental plots at a height of 2 m. Throughout the trial, uniform nutrient and irrigation management protocols were implemented. Specifically, sprinkler irrigation was administered 2–3 times weekly from November 2022 to January 2023, while irrigation was suspended from May to October in both 2022 and 2023. Fertilization was conducted every 2–3 months post‐planting, applying a compound blend of potassium sulfate (400 kg/ha), calcium magnesium phosphate fertilizer (1500 kg/ha), and urea (750 kg/ha).

### 2.2 Sample Collection and Essential Oil Extraction

#### 2.2.1 Lemongrass Sample Collection

In October 2023, within each sampling plot, 30 lemongrass plants were randomly selected. Plants with tillers bearing five fully developed leaves were manually harvested by cutting 2–3 cm above the base of the lemongrass plants. Following natural drying for 1 week, the harvested material was subsequently divided into three groups, each containing 10 plants. For each group, leaf and stem sections were separately excised into 3–5 cm segments. Before stalk elongation, no true stem is present; the base consists of a pseudostem formed by tightly wrapped leaf sheaths. At this stage, tissues were partitioned into “leaf blade” and “pseudostem (leaf‐sheath fraction)”. After stalk emergence, the newly elongated true stem together with its surrounding sheaths was treated as an integrated unit and designated “stem (leaf sheath and stem)”. To ensure consistency, all samples were harvested at a uniform height of 2–3 cm above the soil surface. The 10 plant replicates per group were then pooled to create composite samples representing stems (the remaining parts excluding the leaves) and leaves, respectively.

#### 2.2.2 Lemongrass Essential Oil Extraction

Essential oils were extracted from lemongrass stems and leaves under different treatments using steam distillation. The protocol was as follows: 50 g of lemongrass samples were weighed, fragmented, and transferred into a distillation flask. A 1:10 mass‐to‐liquid ratio was maintained by adding 500 mL of distilled water. Distillation was performed for 4 h at a power setting of 800 W. Post‐distillation, the extracted oil was dehydrated using anhydrous sodium sulfate to remove residual moisture. The oil quality was subsequently assessed by measuring yield, chromaticity, and refractive index. Finally, the volatile components of lemongrass essential oil were analyzed via Headspace Solid‐Phase Microextraction (HS‐SPME) coupled with Gas Chromatography–Mass Spectrometry (GC–MS) to identify key aromatic constituents.

### 2.3 Determination of Physical Properties of Lemongrass Oil

#### 2.3.1 Refractive Index

The refractive index serves as a critical physical constant for differentiating volatile oils. Measurements were conducted under controlled conditions at 20°C using an Abbe refractometer (WYA‐2 W, Shanghai Yidian Physical Optical Instrument Co. Ltd., Shanghai, China). For each lemongrass oil sample, two droplets were deposited on the refractometer prism, followed by securing the prism closure. After allowing the instrument to stabilize for approximately 10 min, refractive index values were recorded from the digital display. Repeat the measurement 3 times for each group of samples.

#### 2.3.2 Oil Yield

The oil yield was determined by measuring the ratio of the volume of lemongrass essential oil extracted via steam distillation to the mass of the stem or leaf tissue samples. The calculation formula is as follows:

$$\begin{array}{cc} \text{Oil Yield}\;\left(\%\right)= & (\text{Volume of Distilled Essential Oil}/\\ & \text{Mass of Lemongrass Sample})\times 100\% \end{array}$$

#### 2.3.3 Chromatic Aberration

The L*, a*, and b* values of lemongrass essential oil were determined using a colorimeter (NH‐310, 3enshi Technology Co. Ltd., Guangdong, China), and the CIE Lab color space was employed to accurately characterize its color differences. This method is based on three key parameters: lightness (L), red‐green axis (a), and yellow‐blue axis (b). The lightness parameter (L) ranges from 0 (black) to 100 (pure white), representing the perceived brightness of the color. The red‐green axis (a) spans values between −128 and +127, where positive values indicate a shift toward red and negative values toward green. Similarly, the yellow‐blue axis (b) also ranges from −128 to +127, with positive values denoting a yellow hue and negative values a blue hue. The total color difference (Δ*E*) was calculated using the following formula:

$$\Delta E=\surd \left(\Delta L^{2}+\Delta a^{2}+\Delta b^{2}\right)$$

where Δ*L*, Δ*a*, and Δ*b* represent the differences in the *L*, *a*, and *b* values between two compared samples.

### 2.4 Determination of Volatile Components of Lemongrass Oil

#### 2.4.1 Pre‐Treatment of Lemongrass Oil Samples

Lemongrass samples were immediately flash‐frozen in liquid nitrogen post‐collection and stored at −80°C to inhibit enzymatic activity. Prior to analysis, the samples were cryogenically ground into powder using liquid nitrogen. A 1 g (or 1 mL) aliquot of the powdered material was transferred into a 20 mL headspace vial (Agilent, Palo Alto, CA, USA), followed by the addition of saturated NaCl solution to quench residual enzymatic reactions. The vials were hermetically sealed using crimp caps fitted with TFE‐silicone septa (Agilent).

#### 2.4.2 HS‐SPME/GC–MS Analysis

The volatile components were extracted using solid‐phase microextraction (SPME) (SPME Arrow, CTC Analytics AG, Switzerland) method. The sample was maintained at a constant temperature of 60°C and agitated for 5 min. A 120 μm DVB/CWR/PDMS fiber was then inserted into the headspace vial for extraction for 15 min. Subsequently, the fiber was desorbed at 250°C for 5 min, followed by GC–MS separation and identification. Prior to sampling, the fiber was conditioned in a Fiber Conditioning Station at 250°C for 5 min.

The volatile components were analyzed by gas chromatography–mass spectrometry (GC–MS) (Agilent 8890‐7000D，Agilent Technologies，USA) under the following conditions: a DB‐5MS capillary column (30 m × 0.25 mm × 0.25 μm; Agilent J&W Scientific, Folsom, CA, USA) was used with high‐purity helium (≥ 99.999%) as the carrier gas at a constant flow rate of 1.2 mL/min. The injector temperature was set to 250°C in splitles mode, with a solvent delay time of 3.5 min. The oven temperature program comprised an initial hold at 40°C for 3.5 min, followed by a ramp at 10°C/min to 100°C, then a second ramp at 7°C/min to 180°C, and finally a rapid increase at 25°C/min to 280°C, where it was held for 5 min. Mass spectrometry parameters were configured as follows: Electron ionization (EI) source maintained at 230°C, quadrupole temperature at 150°C, and MS interface temperature at 280°C. Ionization was performed with 70 eV electron energy. Analytical detection employed selected ion monitoring (SIM) mode, with precise qualitative and quantitative ion scanning executed in accordance with GB 23200.8–2016.

#### 2.4.3 Identification of Volatile Components

In the analysis of volatile components, qualitative analysis is performed by comparing the mass spectra of detected compounds with those from the NIST library, combined with retention times and characteristic ion fragments for identification. For compounds without matching standards in the library, supplementary identification is conducted by referring to data from relevant literature.

#### 2.4.4 Membership Function Analysis of Key Quality Indicators for Lemongrass Oil

The calculation formula for the membership function is as follows:

$$U\left(X_{i}\right)=\left(X_{i}-X_{\min}\right)/\left(X_{\max}-X_{\min}\right)$$

In this equation, *U*(*X*_i_) represents the membership function value, where *U*(*X*_i_) *∈* [0, 1]. *X*_i_ denotes the measured value of indicator iii, while *X*_min_ and *X*_max_ refer to the minimum and maximum values of indicator *i* under each treatment, respectively. The average membership value of each indicator serves as the comprehensive evaluation value.

### 2.5 Statistical Analysis

In this study, two‐way ANOVA was used to analyze the differences in oil yield, refractive index, chromatic aberration, and main component contents of lemongrass under different light conditions and plant parts. Principal Component Analysis (PCA) was employed to evaluate the characteristics and differences of major metabolite categories in lemongrass essential oils across treatments. Molecular network analysis was conducted to identify correlations among refractive index, oil yield, chromatic aberration, and metabolite categories. Correlation analysis was used to determine the relationships among major volatile components in lemongrass oil. Data processing and analysis were performed using software such as SPSS 23.0 and SAS V8, while Origin 2021b and Cytoscape V3.8.2 were utilized for graphing and visualization.

## 3 Result

### 3.1 Comparative Study of the Differences in Physicochemical Properties of Lemongrass Leaves and Stems Under Different Treatments

The oil yield, refractive index, and chromatic aberration of CL essential oil were 0.58%, 1.49, and 74.92, respectively; those of CS essential oil were 0.19%, 1.49, and 74.85, respectively. For HL and HS essential oils, the corresponding values were 0.62%, 1.49, 74.76, and 0.36%, 1.49, 74.85, respectively. Results from two‐way ANOVA showed that the refractive index, oil yield, and chromatic aberration of lemongrass essential oils exhibited no significant differences between natural light and shading treatments. However, significant differences in oil yield were observed between plant parts, with the oil yield of CL significantly higher than that of CS by 54.48% (*p* < 0.05). No significant differences in chromatic aberration or refractive index were found between stem‐derived and leaf‐derived essential oils (Tables 1 and 2). Therefore, the oil yield of lemongrass leaf essential oil was significantly higher than that of stem essential oil, but chromatic aberration and refractive index exhibited no differences. These results indicate that extracting essential oil from the leaves is more efficient, and there are no significant differences in quality between leaf and stem essential oils.

**TABLE 1:** Two‐way ANOVA (*F* values) of the physical characteristics of lemongrass essential oils between normal light and shading treatments (*R*) and between leaf and stem essential oils (*P*).

Treatment	Oil yield (%)	Refractive index	Chromatic aberration
*P*	9.16*	0.50	3.17
*R*	0.18	0.50	0.40
*P* × *R*	0.02	4.50	0.03

* *p* < 0.05.

**TABLE 2:** Differences in oil yield, refractive index, and chromatic aberration of essential oils from lemongrass leaves (L) and stems (S) under normal light treatment (C) and shading treatment (H).

Characterization	C		H	
	L	S	L	S
Oil yield (%)	0.58 ± 0.07a	0.19 ± 0.04b	0.62 ± 0.13a	0.36 ± 0.17ab
Refractive index	1.49 ± 0.01a	1.49 ± 0.01a	1.49 ± 0.00a	1.49 ± 0.00a
Chromatic aberration	74.92 ± 0.30a	74.85 ± 0.14a	74.76 ± 0.16a	74.85 ± 0.23a

*Note:* Different letters indicate significant differences in the physical characteristic indicators of essential oils between lemongrass stems and leaves, while the same letters indicate no significant differences.

### 3.2 Comparison of Content Differences in Metabolite Categories Between Lemongrass Leaves and Stems Under Different Treatments

**FIGURE 1:**
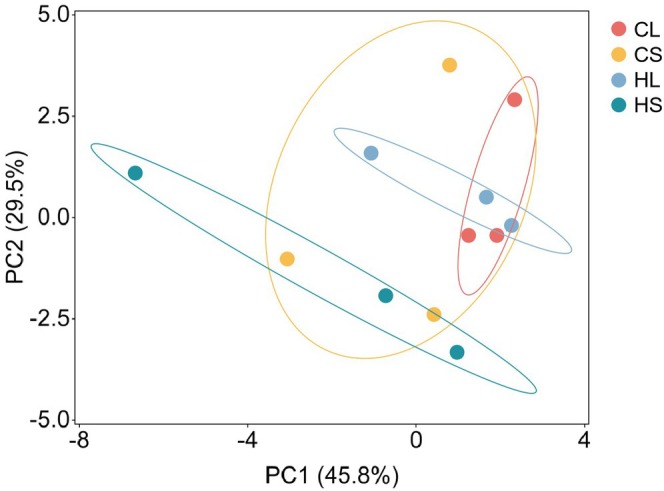
PCA analysis of the main volatile components in the essential oils from lemongrass leaves (L) and stems (S) under normal light (C) and shading (H) treatments.

PCA accounted for 75.30% of the influence of different light conditions and plant parts on the aroma components of lemongrass. Specifically, PC1 explained 45.80% and PC2 accounted for 29.50%. The categories of volatile compounds in the HS treatment were significantly different from those in the HL and CL treatments. The effects of other treatments on the aroma components were not significant, indicating that natural light did not change the categories of volatile components in lemongrass stem and leaf essential oils (Figure 1). However, shading significantly altered the categories of volatile components in lemongrass stem and leaf essential oils. Notably, the categories of volatile components in lemongrass stem and leaf essential oils were not sensitive to changes in light conditions.

**FIGURE 2:**
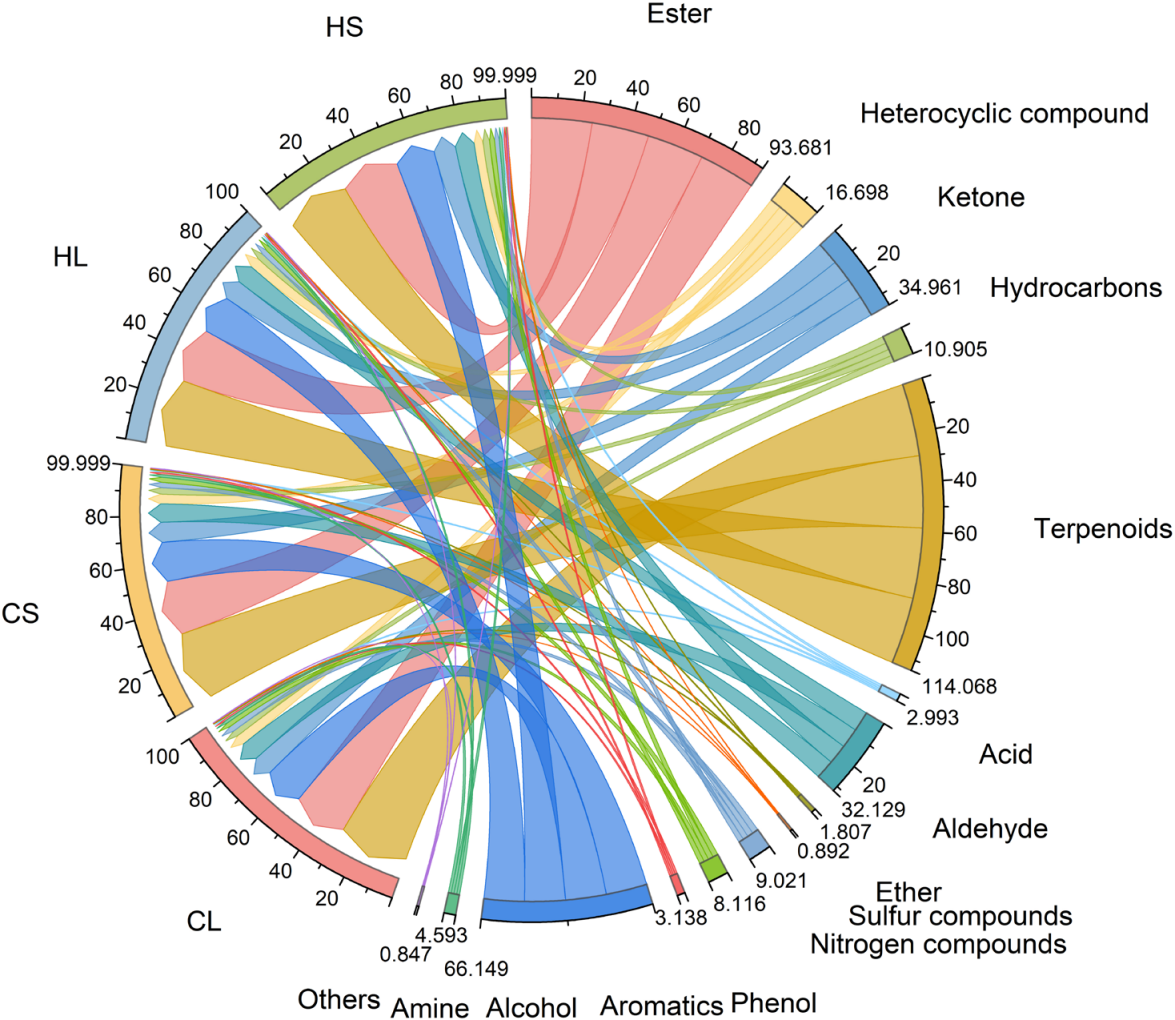
Differences in the content of metabolite categories in essential oils from lemongrass leaves (L) and stems (S) under normal light treatment (C) and shading conditions (H).

Using HS‐SPME (headspace solid‐phase microextraction) and gas chromatography–mass spectrometry (GC–MS), the main component categories and contents of lemongrass oil from leaf and stem parts under natural light and shading treatments were determined. A total of 14 metabolite categories were detected, including ester, heterocyclic compounds, hydrocarbons, terpenoids, acid, aldehyde, ether, sulfur compounds, ketone, nitrogen compounds, phenol, aromatics, alcohol, and amine. The two‐way ANOVA results demonstrated that in lemongrass essential oil, the contents of heterocyclic compounds, ethers, and aldehydes in leaf tissues were significantly higher than those in stems by 14.61% (*p* < 0.05), 10.83% (*p* < 0.05), and 1.57% (*p* < 0.05), respectively. Conversely, hydrocarbon content in leaf tissues was significantly lower than in stems by 6.10% (*p* < 0.05), while other compound categories showed no significant differences between stems and leaves. Furthermore, no alterations were observed in the categories or contents of essential oil compounds before and after shading treatment (Table 3, Figure 2). These findings indicate that shading does not influence the compositional profile or quantitative levels of essential oil compounds in lemongrass, suggesting that variations in radiation intensity do not modify the chemical diversity or abundance of compounds in either stems or leaves. However, the overall compound content in leaf‐derived essential oil was superior to that of stem‐derived oil.

**TABLE 3:** Two‐way ANOVA (*F* values) of major component categories in lemongrass essential oils between normal light and shading treatments (*R*) and between leaf and stem essential oils (*P*).

Treatment	Ester	Heterocyclic compound	Ketone	Hydrocarbons	Terpenoids
*P*	1.94	9.85*	0.45	6.12*	1.36*
*R*	2.68	0.16	1.58	0.99	4.02
*P* × *R*	1.30	3.84	0.18	0.05	0.73

Treatment	Acid	Aldehyde	Ether	Sulfur compounds	Nitrogen compounds
*P*	0.99	8.75*	8.75*	0.09	3.12
*R*	1.92	4.55	4.55	0.05	1.10
*P* × *R*	0.28	1.41	1.40	0.03	0

Treatment	Phenol	Aromatics	Alcohol	Amine	Others
*P*	4.19	2.13	0.19	0.68	0.12
*R*	0.01	0.13	0.31	0	0.52
*P* × *R*	0.01	0.04	0	0.04	0.01

* *p* < 0.05.

### 3.3 Content Differences of Major Volatile Components in Lemongrass Leaves and Stems Under Different Treatments

This study systematically analyzed the chemical composition of lemongrass essential oil using Headspace Solid‐Phase Microextraction (HS‐SPME) coupled with Gas Chromatography–Mass Spectrometry (GC–MS) and detected a total of 994 chemical components. Among the 994 detected components, 110 major components were screened out, accounting for approximately 90% of the total content. The results showed that 56 major components exhibited significant differences between leaves and stems, with 47 components having significantly higher contents in leaves than in stems (Table A1).

**FIGURE 3:**
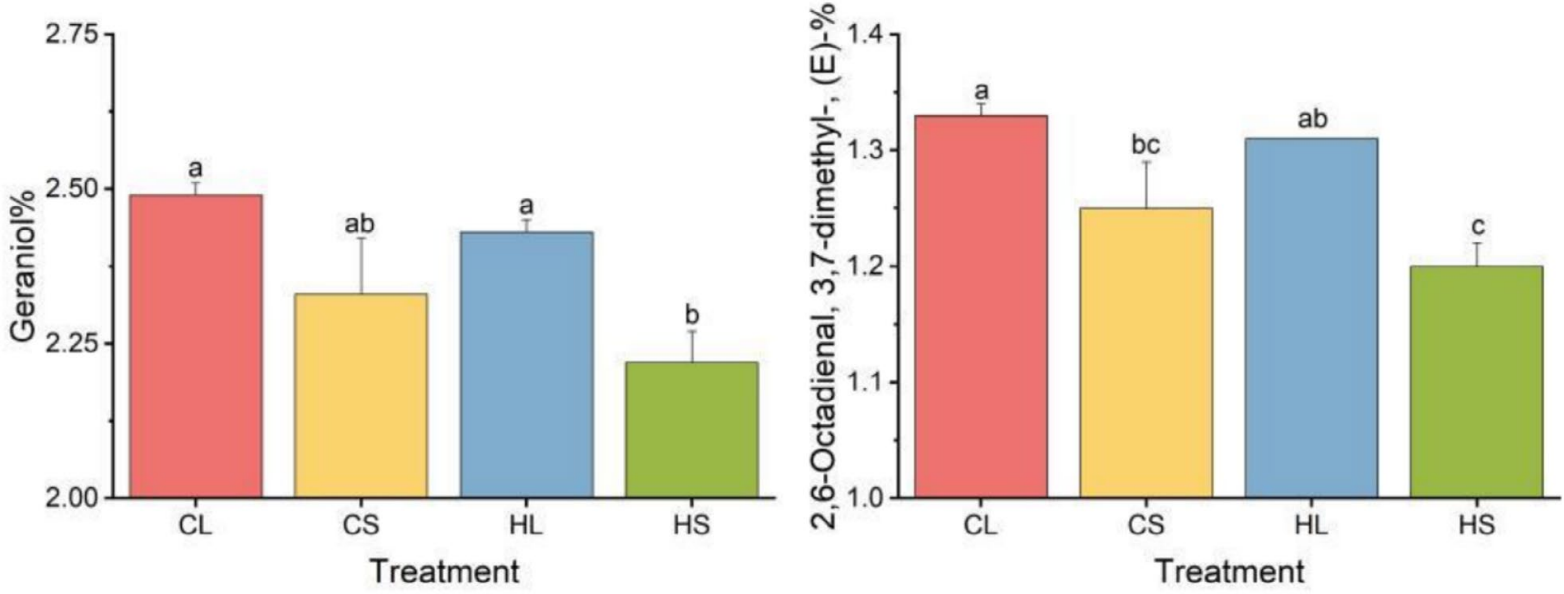
Comparison of the differences in the content of 2 major metabolites in the essential oils of lemongrass leaves (L) and stems (S) under normal light treatment (C) and shading conditions (H). *Note:* In the figure, for letters a, b, c, and d, the same letter indicates no significant difference, while significant differences exist between different letters.

This study conducted further two‐way ANOVA analysis, revealing that the content of geraniol and (E)‐3,7‐dimethyl‐2,6‐octadienal—the most critical constituents in lemongrass essential oil—showed no significant differences across varying radiation treatments, indicating minimal shading effects on these primary components. However, geraniol and (E)‐3,7‐dimethyl‐2,6‐octadienal exhibited significantly higher concentrations in leaf tissues compared to stems by 8.64% and 7.20%, respectively (*p* < 0.01; Figure 3, Table 4). Although geraniol and citral generally displayed no significant variations under different radiation conditions, both compounds demonstrated a decreasing trend following shading treatment. These findings collectively suggest that the majority of major constituents in leaf‐derived essential oil are present at markedly higher levels than in stem‐derived oil, while shading treatment exerts no significant influence on the principal chemical composition of lemongrass essential oil.

**TABLE 4:** Two‐way ANOVA (*F* values) of major aroma components in lemongrass essential oils between normal light and shading treatments (*R*) and between leaf and stem essential oils (*P*).

Treatment	Geraniol	2,6‐octadienal, 3,7‐dimethyl‐, (E)‐
*P*	11.998**	16.3**
*R*	2.527	1.939
*P* × *R*	0.239	0.182

** *p* < 0.01.

### 3.4 Comprehensive Evaluation of Lemongrass Essential Oils From Different Parts Under Different Treatments

In this study, 29 components with relative contents greater than 1% were screened to evaluate the key quality indicators of lemongrass essential oil. Membership function values for essential oils from lemongrass leaves and stems under shading and normal light treatments were calculated, followed by the calculation of their average values. A higher average membership function value (comprehensive evaluation value) indicated better quality of the main components in the treatment or plant part. The ranking of comprehensive evaluation values for major components across treatments showed that the average membership degrees of main components in leaf and stem essential oils under natural light treatment were higher than those under shading treatment. Additionally, the average membership degree of main components in leaf essential oils was higher than that in stem essential oils, suggesting that lemongrass essential oil quality was superior under natural light treatment (Table 5).

**TABLE 5:** Membership function values of volatile organic compounds of essential oils of lemongrass leaves (*L*) and stems (*S*) under normal light treatment (*C*) and shading conditions (*H*).

Main volatile components	Classification	CL	CS	HL	HS
Trans‐Geranic acid methyl ester	Ester	1.000	1.000	1.000	1.000
2,6‐Octadienoic acid, 3,7‐dimethyl‐, methyl ester	Ester	1.000	0.994	0.997	0.977
Undecanol‐5	Alcohol	0.957	1.064	0.922	0.884
1‐Undecyn‐4‐ol	Alcohol	0.408	0.369	0.398	0.305
Ethanol, 2‐(3,3‐dimethylcyclohexylidene)‐, (Z)‐	Alcohol	0.408	0.368	0.398	0.305
4‐Hexen‐1‐ol, 5‐methyl‐2‐(1‐methylethenyl)‐, acetate	Ester	0.408	0.368	0.398	0.305
4‐Isopropyl‐1,3‐cyclohexanedione	Ketone	0.407	0.368	0.397	0.303
7‐Oxabicyclo[4.1.0]heptan‐2‐one, 3‐methyl‐6‐(1‐methylethyl)‐	Terpenoids	0.407	0.368	0.396	0.303
7‐Oxabicyclo[4.1.0]heptan‐2‐one, 6‐Methyl‐3‐(1‐methylethyl)‐	Ketone	0.407	0.368	0.396	0.303
**Geraniol**	Terpenoids	0.407	0.368	0.396	0.303
2,6‐Octadien‐1‐ol, 3,7‐dimethyl‐	Terpenoids	0.406	0.368	0.397	0.302
2,6‐Octadienenitrile, 3,7‐dimethyl‐, (Z)‐	Nitrogen compounds	0.344	0.281	0.306	0.216
10‐Undecenal	Aldehyde	0.210	0.270	0.232	0.301
3‐Cyclohexene‐1‐ethanol, .beta., 4‐dimethyl‐	Alcohol	0.158	0.201	0.154	0.204
Carvone oxide, trans‐	Terpenoids	0.170	0.146	0.171	0.121
1‐Cyclohexene‐1‐carboxaldehyde, 4‐(1‐methylethyl)‐	Terpenoids	0.166	0.149	0.163	0.123
Ethanone, 1‐(2,4‐dihydroxyphenyl)‐	Ketone	0.048	0.104	0.044	0.100
Phenol, 4‐ethyl‐2‐methoxy‐	Phenol	0.047	0.102	0.042	0.098
2,4‐Decadien‐1‐ol	Alcohol	0.044	0.043	0.048	0.039
**2,6‐Octadienal, 3,7‐dimethyl‐, (E)‐**	Terpenoids	0.044	0.043	0.048	0.039
(2,2,6‐Trimethyl‐bicyclo[4.1.0]hept‐1‐yl)‐methanol	Alcohol	0.044	0.043	0.047	0.039
2‐Decenal, (Z)‐	Aldehyde	0.043	0.038	0.042	0.037
1‐(Furan‐2‐yl)‐2‐methylpentan‐1‐one	Heterocyclic compound	0.033	0.032	0.038	0.026
Isobornyl formate	Ester	0.029	0.027	0.031	0.018
3,6‐Octadien‐1‐ol, 3,7‐dimethyl‐, (Z)‐	Terpenoids	0.030	0.021	0.010	0.005
OxiranecarboxAldehyde, 3‐methyl‐3‐(4‐methyl‐3‐pentenyl)‐	Aldehyde	0.032	0.008	0.013	0.007
(−)‐Cis‐Isopiperitenol	Terpenoids	0.000	0.000	0.000	0.000
Average		0.289	0.278	0.279	0.245
Rank		1	3	2	4

*Note:* The two bolded components namely Geraniol and 2,6‐Octadienal, 3,7‐dimethyl‐, (E)‐, are the data of the two components in Figure 3.

### 3.5 Correlations Between Physicochemical Properties and Metabolite Categories in Lemongrass

**FIGURE 4:**
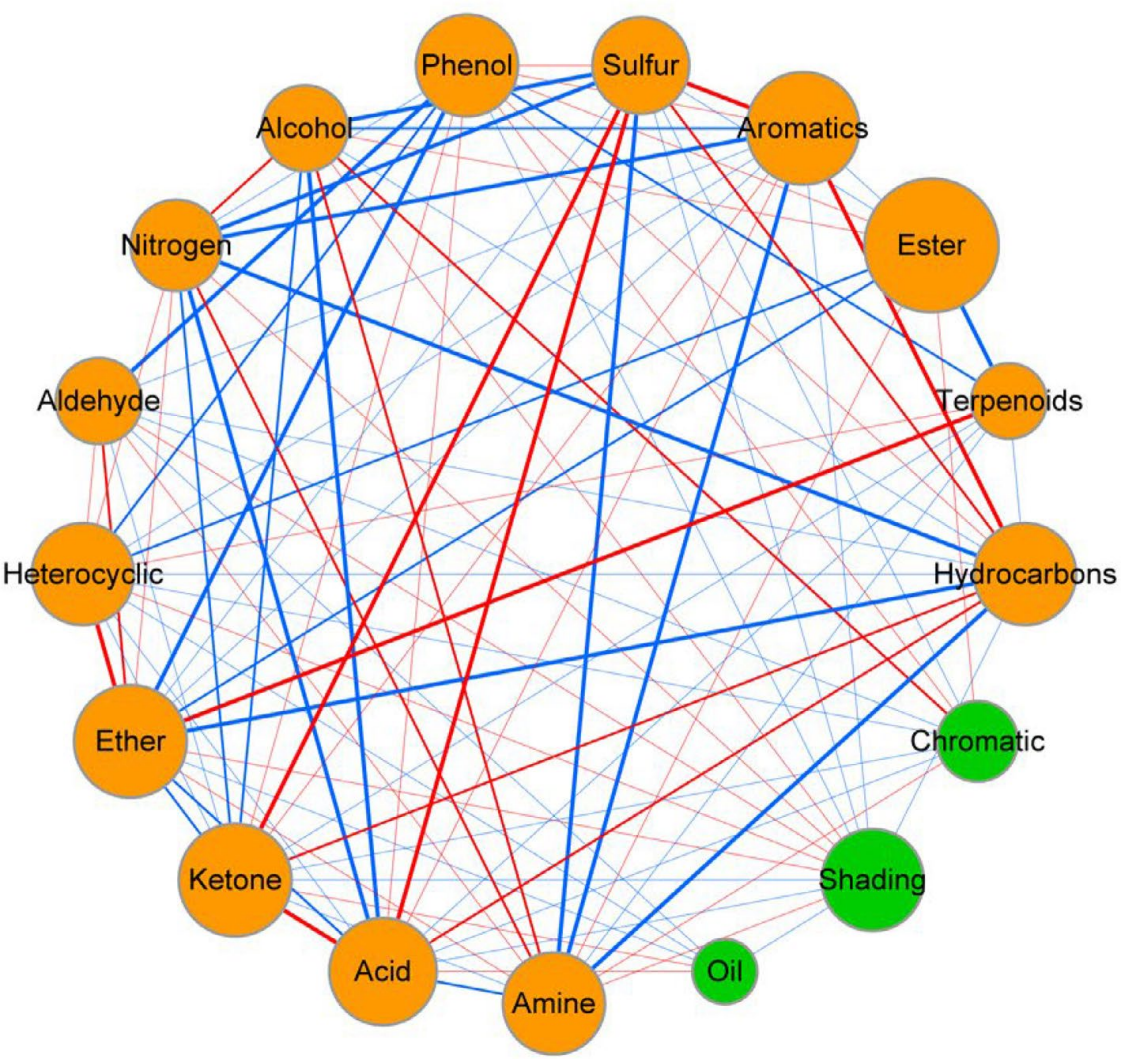
Network interaction diagram of refractive index, oil yield, chromatic aberration, and major components in lemongrass oil. Lines between circles denote correlations, with red lines indicating positive correlations and blue lines indicating negative correlations. The size of the points represents the content level of the indicators, while the thickness of the lines indicates the strength of the correlation. Each circle represents an indicator (*p* < 0.05).

The average network connectivity (avgK), average clustering coefficient (avgCC), average path length (APL), and graph density of the network linking physicochemical properties and volatile metabolite categories in lemongrass oil were 4.59, 0.38, 2.61, and 0.16, respectively (Table 6). Correlation analysis of the network interaction diagram between refractive index, oil yield, chromatic aberration, and major components of lemongrass essential oil showed strong negative correlations among the indicators, with particularly evident negative correlations between major components (Table 6, Figure 4), indicating strong antagonistic effects among these components. Significant negative correlations (*p* < 0.05) were observed between Nitrogen and Sulfur, Acid, Aromatics, Hydrocarbons; between Amine and Sulfur, Aromatics, Hydrocarbons; and between Phenol and Aldehyde, Heterocyclic, Ether, Terpenoids, suggesting obvious antagonistic effects among these substances. In contrast, significant positive correlations (*p* < 0.05) were found between Sulfur, Ketone, and Acid; between Nitrogen, Alcohol, and Amine; and between Aromatics and Hydrocarbons, indicating prominent synergistic effects among these components.

**TABLE 6:** Topological properties of the correlation network between major metabolites and physicochemical properties in lemongrass essential oil.

Network metrics	Relative contents
Number of nodes	16
Number of edges	98
Number of positive correlations	42
Number of negative correlations	56
Percentage of the positive link (P%)	42.86%
P% from physicochemical properties to volatile substance components	44.44%
P% among volatile substance components	40.85%
Average connectivity (avgK)	4.59
Average clustering coefficient (avgCC)	0.38
Average path length (APL)	2.61
Graph density	0.16

### 3.6 Correlations of Major Metabolites in Lemongrass

**FIGURE 5:**
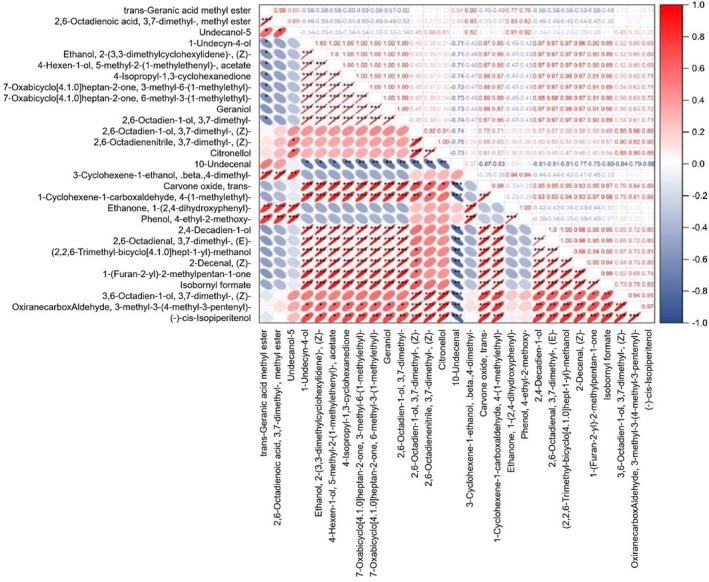
Correlation heatmap of major chemical substances in lemongrass oil. The intensity of color and the flatness of ellipses represent the strength of the correlation, where red indicates positive correlation and blue indicates negative correlation. Darker colors signify stronger correlations, and flatter ellipses indicate stronger correlations. “*” denotes significant correlation at *p* < 0.05, “**” denotes extremely significant correlation at *p* < 0.01, and “***” denotes extremely significant correlation at *p* < 0.001.

Based on Pearson correlation coefficient analysis, the interrelationships among 29 major components of lemongrass oil were revealed. Results showed that most major components exhibited extremely significant positive correlations, while only a few showed significant negative correlations. Specifically, the main volatile compounds Geraniol and (E)‐3,7‐dimethyl‐2,6‐octadienal demonstrated extremely significant positive correlations with trans‐Carvone oxide (*r* = 0.88, *p* < 0.001; *r* = 0.95, *p* < 0.001), 4‐(1‐methylethyl)‐1‐cyclohexene‐1‐carboxaldehyde (*r* = 0.87, *p* < 0.001; *r* = 0.93, *p* < 0.001), 2,4‐decadien‐1‐ol (*r* = 0.97, *p* < 0.001; *r* = 1.00, *p* < 0.001), (Z)‐2‐decenal (*r* = 0.97, *p* < 0.001; *r* = 0.98, *p* < 0.001), and 1‐(furan‐2‐yl)‐2‐methylpentan‐1‐one (*r* = 0.91, *p* < 0.001; *r* = 0.95, *p* < 0.001) (Figure 5). Geraniol and (E)‐3,7‐dimethyl‐2,6‐octadienal also showed extremely significant positive correlation with each other (*r* = 0.97, *p* < 0.001). These findings suggest that these chemical components of lemongrass oil share common metabolic pathways during synthesis and potentially act together in contributing to the aroma and flavor of lemongrass oil. In contrast, 10‐undecenal exhibited significant negative correlations with 22 of the major components (*p* < 0.05) (Figure 5), indicating potential competitive relationships during synthesis and potentially mutually offsetting effects on the aroma and flavor of lemongrass oil.

## 4 Discussion

### 4.1 Effects of Radiation Intensity Variations on the Physicochemical Properties of Lemongrass Stem and Leaf Essential Oils

Refractive index and chromatic aberration have been established as the core indicators of lemongrass essential oil purity and stability, while oil yield directly affects extraction process efficiency and economic conversion benefits. The refractive index of essential oil correlates with its chemical composition; specifically, a higher refractive index indicates a higher concentration of active components and better purity (Lia Umi et al. 2024; Masyita et al. 2022). Dynamic changes in refractive index during storage serve as a sensitive indicator of oxidation reaction progression and chemical stability (Ganosi et al. 2023; Khodier 2002). Previous studies have established that high‐quality lemongrass essential oil is characterized by a refractive index falling within the range of 1.4830–1.4890 at 20°C (Edeh and Okpo 2023; Heritage 2016; Mieso et al. 2020). Results from this study demonstrated that the refractive index of tested oils from lemongrass stems and leaves under different radiation intensity treatments did not differ significantly (Table 1), consistent with previous findings. Given that high‐quality lemongrass essential oil is characterized by a specific refractive index range, the stability of the refractive index across treatments validates the reliability of refractive index as a rapid indicator of essential oil quality, while also providing empirical evidence for optimizing the storage conditions of lemongrass essential oil.

Chromatic aberration analysis serves as a critical method for evaluating oxidative damage or foreign substance contamination in lemongrass essential oil. The color of essential oil is influenced by extraction processes, storage environments, compound compositions, and geographical origin conditions (Mehrotra et al. 2023). Previous studies have demonstrated that low‐quality lemongrass essential oil is typically colorless, whereas high‐quality oil exhibits a light yellow color (Tran et al. 2021). Under these experimental conditions, no significant differences in lemongrass essential oil color were observed across different treatments (Tables 1 and 2), indicating that chromatic aberration displays limited sensitivity to changes in light intensity within the tested range.

The oil yield of plant essential oils is regulated by multiple factors, with the synthesis efficiency of plant essential oils influenced by the synergistic effects of organ specificity, developmental stages, and environmental factors (Ilić et al. 2022; Mehalaine and Chenchouni 2021; Šunić et al. 2023). Previous studies have demonstrated that different tissues such as leaves, flowers, stems, and seeds in plants exhibit distinct metabolic patterns through differential expression of terpene synthase genes. For example, essential oils in mint (*Mentha* spp.) are primarily concentrated in leaves (Sara et al. 2024), while the accumulation of leaf essential oils remains at a low level in wild fennel (Šunić et al. 2023). In *Citrus aurantium* “*changshanhuyou*,” essential oils are mainly enriched in peel tissues where the oil gland layer is well‐developed (Han et al. 2024). Radiation conditions exert a significant regulatory effect on plant essential oil biosynthesis. Previous studies demonstrated that lemon balm ( *Melissa officinalis* ) grown under shaded conditions had substantially higher essential oil content (0.45 mL/100 g) compared to unshaded plants (0.21 mL/100 g) (Lalević et al. 2023). While our findings suggest potential shading‐induced influences on lemongrass essential oil synthesis and accumulation patterns (Tables 1 and 2), they also indicate that lemongrass may possess flexible resource allocation mechanisms. These adaptive strategies enable both leaf and stem tissues to efficiently synthesize and accumulate essential oil components under different radiation intensities, possibly through differential partitioning of metabolic resources across plant organs under light stress adaptation. The significantly higher oil yield in leaves than in stems under normal light treatment suggests that this phenotypic plasticity in lemongrass may involve chloroplast structural remodeling and optimization of photosynthate transport mechanisms, serving as a mechanism to mitigate low light stress by adjusting carbon flux allocation. The environmental adaptation mechanism of lemongrass plant essential oil biosynthesis revealed in this study not only confirms the co‐evolutionary relationship between secondary metabolite synthesis and ecological adaptation but also provides a theoretical basis for cultivating aromatic plants through microenvironmental regulation to directionally induce essential oil accumulation directionally in target organs.

### 4.2 Effects of Different Radiation Intensities on Metabolite Classification Categories in Lemongrass Stem and Leaf Essential Oils

Lemongrass essential oil has a complex chemical composition, primarily consisting of terpenoids (notably β‐myrcene), aldehydes (geranial and neral), alcohols (geraniol, nerol, and linalool), along with minor phenolic and ketonic compounds. These constituents collectively endow the essential oil with distinctive aromatic properties, antimicrobial activity, and significant impacts on its bioactivity and physicochemical characteristics (Gautam and Agrawal 2017; Hamad et al. 2017; Lammari et al. 2021; Sarah et al. 2023). Previous studies on *wild fennel* have revealed significant differences in the composition and content of essential oils among different plant parts (leaves, stems, and fruits), the essential oils from its leaves and stems contain a higher proportion of monoterpenoids, whereas those from fruits may contain more sesquiterpenoids (Khalid 2024). In *Platycladus orientalis* , the essential oil from its fruits has a higher content of sesquiterpenoids, showing a significant difference from that in the leaves where monoterpenoids are the main components (Xie and Liu 2024). In this study, the contents of heterocyclic compounds, ethers, and aldehydes in lemongrass leaf essential oil were significantly higher than those in the lemongrass stem essential oil, while the hydrocarbon content was significantly lower (Figure 2). This may be ascribed to the greater sensitivity of lemongrass leaves to changes in light radiation, which are closely related to their light‐responsive metabolic regulatory mechanisms (Madheshiya et al. 2024).

Radiation conditions serve as critical environmental signals that regulate crop metabolite profiles by altering photosynthetic characteristics. For instance, under shaded conditions, the content of piperitenone oxide, a major constituent in peppermint essential oil, increased from 52.6% in unshaded plants to 64.8%, while that of 1,8—cineole decreased from 25.9% to 16.3% (Lalević et al. 2023). However, our experimental results demonstrate no significant differences in the chemical composition of lemongrass essential oil between shaded and natural treatments. It is probable shading conditions, precursor metabolites in lemongrass that were originally utilized for synthesizing specific essential oil components were redistributed to other biosynthetic pathways, resulting in the generation of different essential oil components. This metabolic adjustment enabled the overall chemical composition of the essential oil to maintain relative stability and balance. Lemongrass may maintain product metabolic homeostasis through the redistribution of precursor substance flux. For example, intermediates in the mevalonate pathway might be channeled to other terpenoid biosynthetic branches, thereby compensating for changes in specific components to a certain extent (Samsami and Maali‐Amiri 2024).

### 4.3 Effects of Different Radiation Intensities on Key Metabolites in Lemongrass Stem and Leaf Essential Oils

The major chemical components of lemongrass essential oil include geranial (27%–45%), neral (20%–33%), β‐myrcene (0.14%–11.41%), linalool (0.2%–5%), and geraniol (1%–13%) (Brügger et al. 2019; Du et al. 2023; Hamad et al. 2017). These components collectively contribute to the unique aroma and diverse biological activities of lemongrass essential oil, such as antibacterial, antioxidant, anti‐inflammatory, sedative, and hypoglycemic properties (Brügger et al. 2019; Mukarram et al. 2021). Results of this study revealed that the contents of the key quality components geraniol and (E)‐3,7‐dimethyl‐2,6‐octadienal in lemongrass leaves were 8.64% and 7.20% higher than those in lemongrass stems, respectively (*p* < 0.01) (Table 3, Figure 3). This suggests that the content of active components in lemongrass leaf essential oil is higher than that in stem essential oil. Moreover, the significant differences in the content of major components of lemongrass essential oil among different plant parts can be mainly ascribed to the functional division of metabolism among organs. Secondary metabolites in lemongrass are mainly synthesized and accumulated in lemongrass leaves. Leaves serve as the primary site of photosynthesis and can supply more energy and precursor substances for the synthesis of essential oil components. In contrast, metabolic activities in stems might be more focused on supporting plant structural and transport functions rather than secondary metabolite synthesis (Hemmati Hassan Gavyar and Amiri 2018; Lia Umi et al. 2024; Sharma et al. 2021).

The significant regulatory impact of shading on the chemical composition and biological activity of crop essential oils is mainly accomplished by affecting the plant's photosynthesis and secondary metabolic processes. Previous studies have revealed that shading treatment significantly affects the yield and chemical composition of plant essential oils, and different plants exhibit diverse responses to shading. For example, when subjected to shading conditions, the yield and content of major components of *Damascus rose* essential oil decrease, while the content of carvacrol in *oregano* essential oil increases, and the content of α‐thujone in *sage* essential oil decreases (Rezaei et al. 2019; Şeker et al. 2023). In this study, the contents of the major components geraniol and (E)‐3,7‐dimethyl‐2,6‐octadienal in lemongrass essential oil exhibited no significant differences under different light conditions (Table 3, Figure 3), indicating that shading treatment had minimal impact on the contents of major components in lemongrass essential oil. This can be ascribed to the unique metabolic regulatory mechanism of lemongrass. When photosynthesis is restricted by shading, the plant optimizes carbon source allocation to preferentially maintain the activity of key terpenoid synthesis pathways (such as the MEP pathway), thus ensuring the stable accumulation of major essential oil components. Meanwhile, shading may activate specific defense‐related metabolic pathways to facilitate the synthesis of certain secondary components without disrupting the composition of core essential oil components. This adaptive strategy allows lemongrass to maintain the stability of essential oil chemical composition under low‐light conditions (Kumar et al. 2025; Szepesi 2021).

This study conducted a preliminary analysis of differences in the content of lemongrass essential oil across different plant parts (leaves and stems) and under different light conditions (shading and normal light), and it was found that the essential oil content was higher in leaves than in stems, and light intensity significantly affected the content of essential oil components. However, the study has not comprehensively elucidated the regulatory mechanisms underlying lemongrass essential oil quality changes across different plant parts and light conditions. The study by comparing only normal (full sunlight) conditions with a single shading level (60% light reduction). Such a limited approach restricts the interpretation of lemongrass's response to varying light intensities. For more meaningful and long‐lasting results, in our subsequent study, we will explicitly incorporate a gradient of four shading treatments (20%, 40%, 60% and 80% photosynthetic photon flux reduction) which would allow for a clearer understanding of the plant's physiological and phytochemical adaptation thresholds. In particular, the impacts of competitive and synergistic interactions among major components on lemongrass essential oil quality and the specific metabolic regulatory mechanisms remain to be further investigated. In the future, transcriptomics and metabolomics techniques could be employed to deeply explore the effects of different light conditions and plant parts on lemongrass essential oil biosynthetic pathways, with the aim of better understanding the molecular mechanisms underlying these regulatory phenomena.

## 5 Conclusion

The volatile components in essential oils derived from lemongrass stems and leaves displayed insensitivity to diverse light conditions. The essential oil from lemongrass leaves was more amenable to extraction than that from stems, although no significant differences were detected in their physicochemical properties. The contents of heterocyclic compounds, ethers, and aldehydes in leaf essential oil were significantly higher than those in stem essential oil, whereas the hydrocarbon content showed an opposite trend. The key quality components geraniol and citral had significantly higher contents in leaf essential oil than in stem essential oil, even though shading tended to reduce their levels. Results suggested that the quality of leaf essential oil under normal light conditions was more favorable. Therefore, the adoption of a normal light cultivation model in combination with targeted leaf harvesting, aiming to overcome the traditional inefficient whole—plant extraction approach, provides a theoretical foundation for the in—depth and high—value processing of lemongrass.

## Author Contributions

**Ling Xu:** conceptualization (equal), investigation (equal), project administration (equal), software (equal), writing – original draft (equal), writing – review and editing (equal). **Nan Lu:** investigation (equal), methodology (equal), project administration (equal), resources (equal), software (equal), writing – original draft (equal), writing – review and editing (equal). **Zhuo Feng:** data curation (equal), validation (equal), visualization (equal). **ZiWei Ning:** formal analysis (equal), supervision (equal), validation (equal). **YanLi Huang:** conceptualization (equal), funding acquisition (equal), software (equal), writing – review and editing (equal). **Chun Xie:** software (equal), writing – review and editing (equal).

## Conflicts of Interest

The authors declare no conflicts of interest.

## Data Availability

The following supporting information can be downloaded at: http://www.10.17632/4rn3srhdny.1.

## Appendix A

**TABLE A1:** Comparison of the relative contents of the 56 significantly different major chemical components in lemongrass leaves and stems.

Volatile substance	Classification	CL (mean ± standard deviation)	CS (mean ± standard deviation)	HL (mean ± standard deviation)	HS (mean ± standard deviation)
Geranyl format	Ester	0.578 ± 0.074	0.188 ± 0.042	0.616 ± 0.129	0.608 ± 0.029
1‐Undecyn‐4‐ol	Alcohol	2.492 ± 0.025	2.335 ± 0.085	2.440 ± 0.025	2.234 ± 0.054
Ethanol, 2‐(3,3‐dimethylcyclohexylidene)‐, (Z)‐	Alcohol	2.492 ± 0.025	2.334 ± 0.086	2.438 ± 0.024	2.235 ± 0.053
4‐Hexen‐1‐ol, 5‐methyl‐2‐(1‐methylethenyl)‐, acetate	Ester	2.492 ± 0.025	2.334 ± 0.086	2.438 ± 0.025	2.234 ± 0.054
4‐Isopropyl‐1,3‐cyclohexanedione	Ketone	2.489 ± 0.025	2.334 ± 0.084	2.436 ± 0.023	2.225 ± 0.047
7‐Oxabicyclo[4.1.0]heptan‐2‐one, 3‐methyl‐6‐(1‐methylethyl)‐	Terpenoids	2.489 ± 0.025	2.334 ± 0.085	2.431 ± 0.024	2.226 ± 0.047
7‐Oxabicyclo[4.1.0]heptan‐2‐one, 6‐methyl‐3‐(1‐methylethyl)‐	Ketone	2.488 ± 0.025	2.333 ± 0.085	2.432 ± 0.019	2.223 ± 0.048
Geraniol	Terpenoids	2.487 ± 0.024	2.333 ± 0.085	2.430 ± 0.020	2.224 ± 0.051
2,6‐Octadien‐1‐ol, 3,7‐dimethyl‐	Terpenoids	2.483 ± 0.019	2.333 ± 0.085	2.434 ± 0.023	2.221 ± 0.044
10‐Undecenal	Aldehyde	1.861 ± 0.026	2.005 ± 0.018	1.902 ± 0.068	2.216 ± 0.038
Carvone oxide, trans‐	Terpenoids	1.734 ± 0.026	1.590 ± 0.042	1.704 ± 0.036	1.518 ± 0.037
1‐Cyclohexene‐1‐carboxaldehyde, 4‐(1‐methylethyl)‐	Terpenoids	1.719 ± 0.034	1.601 ± 0.049	1.681 ± 0.035	1.528 ± 0.028
2,4‐Decadien‐1‐ol	Alcohol	1.331 ± 0.009	1.246 ± 0.039	1.309 ± 0.005	1.203 ± 0.024
2,6‐Octadienal, 3,7‐dimethyl‐, (E)‐	Terpenoids	1.331 ± 0.010	1.245 ± 0.039	1.309 ± 0.004	1.202 ± 0.024
(2,2,6‐Trimethyl‐bicyclo[4.1.0]hept‐1‐yl)‐methanol	Alcohol	1.331 ± 0.010	1.245 ± 0.040	1.307 ± 0.005	1.202 ± 0.024
2‐Decenal, (Z)‐	Aldehyde	1.327 ± 0.008	1.230 ± 0.044	1.291 ± 0.004	1.196 ± 0.023
1‐(Furan‐2‐yl)‐2‐methylpentan‐1‐one	Heterocyclic compound	1.297 ± 0.035	1.210 ± 0.053	1.275 ± 0.025	1.150 ± 0.021
Isobornyl formate	Ester	1.284 ± 0.028	1.193 ± 0.050	1.253 ± 0.031	1.121 ± 0.030
OxiranecarboxAldehyde, 3‐methyl‐3‐(4‐methyl‐3‐pentenyl)‐	Aldehyde	1.293 ± 0.009	1.130 ± 0.034	1.196 ± 0.057	1.078 ± 0.081
(−)‐cis‐Isopiperitenol	Terpenoids	1.192 ± 0.014	1.103 ± 0.021	1.154 ± 0.027	1.051 ± 0.050
6‐Undecanol	Alcohol	0.963 ± 0.008	1.039 ± 0.024	0.952 ± 0.013	1.083 ± 0.036
1‐Tridecene	Hydrocarbons	0.951 ± 0.011	1.015 ± 0.034	0.926 ± 0.018	1.042 ± 0.060
(1R, 5S, 6R)‐2,7,7‐Trimethylbicyclo[3.1.1]hept‐2‐en‐6‐yl acetate	Ester	0.777 ± 0.012	0.720 ± 0.012	0.759 ± 0.015	0.689 ± 0.015
Bicyclo[3.1.1]hept‐2‐en‐6‐ol, 2,7,7‐trimethyl‐, acetate, [1S‐(1.alpha.,5.alpha.,6.beta.)]‐	Ester	0.776 ± 0.013	0.717 ± 0.012	0.754 ± 0.017	0.687 ± 0.017
1‐Tridecyne	Hydrocarbons	0.586 ± 0.006	0.621 ± 0.014	0.613 ± 0.021	0.685 ± 0.006
(2E,4Z)‐2,4‐Decadienal	Aldehyde	0.590 ± 0.009	0.603 ± 0.031	0.618 ± 0.022	0.692 ± 0.010
2,4‐Decadienal, (E,E)‐	Aldehyde	0.582 ± 0.005	0.614 ± 0.017	0.589 ± 0.007	0.662 ± 0.018
p‐Menthane‐3,8‐diol, cis‐1,3,trans‐1,4‐	Terpenoids	0.582 ± 0.005	0.616 ± 0.016	0.586 ± 0.007	0.650 ± 0.018
(−)‐Bornyl acetate	Terpenoids	0.572 ± 0.005	0.611 ± 0.018	0.576 ± 0.006	0.641 ± 0.024
(E)‐2‐Decenal	Aldehyde	0.562 ± 0.061	0.460 ± 0.063	0.626 ± 0.013	0.449 ± 0.035
Benzeneacetic acid	Acid	0.470 ± 0.009	0.433 ± 0.011	0.468 ± 0.007	0.419 ± 0.005
Benzene, (butoxymethyl)‐	Ether	0.470 ± 0.009	0.433 ± 0.011	0.468 ± 0.007	0.416 ± 0.009
Benzenemethanamine, N‐(1‐methylethyl)‐	Amine	0.468 ± 0.010	0.423 ± 0.008	0.462 ± 0.005	0.398 ± 0.016
Picolinamide	Heterocyclic compound	0.440 ± 0.0061	0.410 ± 0.011	0.438 ± 0.005	0.398 ± 0.004
Butyramide, 2‐cyano‐2‐ethyl‐	Amine	0.364 ± 0.006	0.387 ± 0.015	0.358 ± 0.004	0.400 ± 0.019
Propanoic acid, 2‐methyl‐, phenylmethyl ester	Ester	0.320 ± 0.002	0.336 ± 0.012	0.322 ± 0.005	0.361 ± 0.013
Phenol, 4‐propyl‐	Phenol	0.343 ± 0.006	0.317 ± 0.008	0.336 ± 0.004	0.303 ± 0.007
Benzenemethanol, .alpha.‐2‐propenyl‐	Alcohol	0.337 ± 0.005	0.310 ± 0.004	0.325 ± 0.012	0.299 ± 0.004
5‐Hepten‐2‐one, 6‐methyl‐	Ketone	0.346 ± 0.018	0.273 ± 0.034	0.394 ± 0.054	0.231 ± 0.060
Phenol, 4‐(2‐propenyl)‐	Aromatics	0.321 ± 0.006	0.296 ± 0.006	0.317 ± 0.006	0.285 ± 0.006
3,5‐Dimethyl‐1‐butylpyrazole	Heterocyclic compound	0.325 ± 0.012	0.277 ± 0.010	0.338 ± 0.003	0.262 ± 0.020
3‐Cyclohexene‐1‐acetAldehyde, .alpha., 4‐dimethyl‐	Aldehyde	0.326 ± 0.014	0.273 ± 0.011	0.336 ± 0.003	0.260 ± 0.022
Cyclohexanone, 5‐methyl‐2‐(1‐methylethenyl)‐	Terpenoids	0.318 ± 0.011	0.273 ± 0.011	0.324 ± 0.003	0.254 ± 0.019
1‐Decanol	Alcohol	0.305 ± 0.005	0.287 ± 0.013	0.299 ± 0.001	0.273 ± 0.006
1H‐Pyrrole‐2,5‐dione, 3‐ethenyl‐4‐methyl‐	Heterocyclic compound	0.306 ± 0.029	0.249 ± 0.032	0.342 ± 0.006	0.249 ± 0.015
Carvenone	Terpenoids	0.301 ± 0.003	0.274 ± 0.009	0.288 ± 0.008	0.259 ± 0.011
3‐p‐Menthen‐7‐al	Aldehyde	0.272 ± 0.009	0.223 ± 0.016	0.275 ± 0.005	0.208 ± 0.027
Butanoic acid, 3‐hexenyl ester, (E)‐	Ester	0.208 ± 0.006	0.184 ± 0.007	0.218 ± 0.001	0.177 ± 0.015
Benzoic acid, 2‐propenyl ester	Ester	0.179 ± 0.003	0.167 ± 0.004	0.177 ± 0.002	0.159 ± 0.004
4, 7, 7‐Trimethylbicyclo[4.1.0]hept‐3‐en‐2‐one	Ketone	0.1581 ± 0.001	0.164 ± 0.006	0.170 ± 0.010	0.185 ± 0.003
Ethanethioic acid, S‐(2‐furanylmethyl) ester	Heterocyclic compound	0.185 ± 0.006	0.160 ± 0.011	0.180 ± 0.005	0.148 ± 0.019
Bicyclo[3.1.1]hept‐2‐ene‐2‐carboxaldehyde, 6,6‐dimethyl‐	Terpenoids	0.134 ± 0.006	0.115 ± 0.004	0.134 ± 0.001	0.107 ± 0.009
5‐propan‐2‐ylbicyclo[3.1.0]hex‐2‐ene‐2‐carbAldehyde	Aldehyde	0.132 ± 0.004	0.114 ± 0.004	0.134 ± 0.001	0.107 ± 0.009
(E)‐2,6‐Dimethylocta‐3,7‐diene‐2,6‐diol	Alcohol	0.131 ± 0.00	0.111 ± 0.004	0.132 ± 0.001	0.104 ± 0.008
Butanoic acid, 3‐hexenyl ester, (Z)‐	Ester	0.128 ± 0.004	0.110 ± 0.004	0.131 ± 0.001	0.104 ± 0.008
2‐Cyclohexen‐1‐one, 3‐methyl‐6‐(1‐methylethenyl)‐, (S)‐	Terpenoids	0.127 ± 0.003	0.109 ± 0.004	0.132 ± 0.001	0.104 ± 0.008
